# Ruptured cystic artery pseudoaneurysm after self‐expandable metal stent placement for malignant biliary obstruction

**DOI:** 10.1002/deo2.304

**Published:** 2023-10-26

**Authors:** Takafumi Mie, Takashi Sasaki, Kiyoshi Matsueda, Takeshi Okamoto, Tatsuki Hirai, Takahiro Ishitsuka, Manabu Yamada, Hiroki Nakagawa, Takaaki Furukawa, Tsuyoshi Takeda, Akiyoshi Kasuga, Masato Ozaka, Naoki Sasahira

**Affiliations:** ^1^ Department of Hepato‐Biliary‐Pancreatic Medicine Cancer Institute Hospital, Japanese Foundation for Cancer Research Tokyo Japan; ^2^ Department of Diagnostic Imaging Cancer Institute Hospital, Japanese Foundation for Cancer Research Tokyo Japan

**Keywords:** endoscopic retrograde cholangiopancreatography, hemobilia, upper gastrointestinal bleeding, endoscopy, transcatheter arterial embolization

## Abstract

We report a case of ruptured cystic artery pseudoaneurysm after self‐expandable metal stent placement for malignant biliary obstruction. A 78‐year‐old woman on palliative care after chemotherapy for unresectable pancreatic head cancer presented with obstructive jaundice. Imaging revealed a dilated common bile duct and an enlarged gallbladder with cystic wall thickening. Endoscopic retrograde cholangiopancreatography was performed and a fully‐covered self‐expandable metal stent was placed in the bile duct, leading to resolution of jaundice. She presented with hematochezia 7 days later. Contrast‐enhanced computed tomography revealed a cystic artery pseudoaneurysm with extravasation of contrast into a blood‐filled gallbladder. Hemostasis was achieved after emergent transcatheter arterial embolization. Rupture of cystic artery pseudoaneurysm should be raised as a differential diagnosis for hemobilia after self‐expandable metal stent placement, particularly in cases accompanied by inflamed gallbladders.

## INTRODUCTION

In patients with unresectable malignant distal biliary obstruction, a self‐expandable metal stent (SEMS) placement is recommended because of its long patency. Common adverse events after SEMS placement include pancreatitis, cholecystitis, cholangitis, and liver abscess. Bleeding after SEMS placement is rare, with a reported frequency of 0%–1.8%.^1^, ^2^ In particular, bleeding from pseudoaneurysms after SEMS placement is rare, with a frequency of 1.2%.^3^ Pseudoaneurysm after SEMS placement often develops in arteries surrounding the common bile duct. Cystic artery pseudoaneurysms, which are very rare, are associated with cholecystitis.^4^ There have been no reports to date on cystic artery pseudoaneurysm rupture after SEMS placement.

Here we report a case of ruptured cystic artery pseudoaneurysm 7 days after SEMS placement for obstructive jaundice with pancreatic cancer, which was successfully treated with transcatheter arterial embolization (TAE).

## CASE REPORT

A 78‐year‐old woman on palliative care after chemotherapy for pancreatic head cancer with hepatic metastases presented with a mild fever of 37.6°C, upper abdominal pain, and jaundice. She had received first‐line modified FOLFIRINOX for 1.5 years and second‐line gemcitabine and nab‐paclitaxel combination chemotherapy for 2 months but was finally placed on palliative treatment. Her medical history included hypertension, diabetes mellitus, deep vein thrombosis, and chemotherapy‐induced peripheral neuropathy. Her medications consisted of amlodipine, rivaroxaban, mirogabalin, and subcutaneous insulin injections. She had no significant family history or history of alcohol consumption or smoking.

**FIGURE 1:**
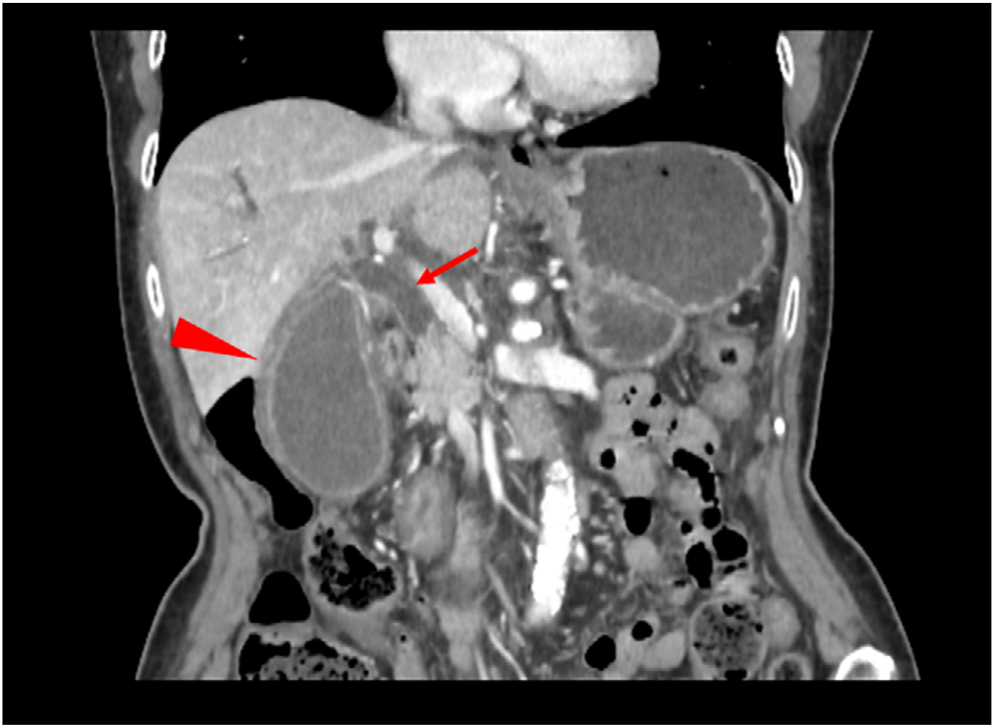
Dilation of the common bile duct (arrow) and an enlarged gallbladder with wall thickening (arrowhead).

Contrast‐enhanced computed tomography revealed a dilated common bile duct and an enlarged gallbladder with wall thickening, suggestive of cholecystitis. No pseudoaneurysms were observed at this time (Figure 1). As there was no obstruction of the cystic duct, endoscopic retrograde cholangiopancreatography (ERCP) was performed for biliary drainage. Endoscopic sphincterotomy was performed and an endoscopic naso‐biliary drainage tube was placed for temporary drainage. Two days later, a 10 mm × 6 cm fully‐covered SEMS (HANAROSTENT Biliary Multi‐hole NEO; Boston Scientific Corp.) was placed, without adverse events. At the time of SEMS placement, the patient was afebrile with a temperature was 36.8°C, and laboratory tests showed a white blood cell count of 5910/μL and C‐reactive protein of 4.32 mg/dL. The resolution of fever and jaundice was confirmed, and she was discharged 7 days later.

**FIGURE 2:**
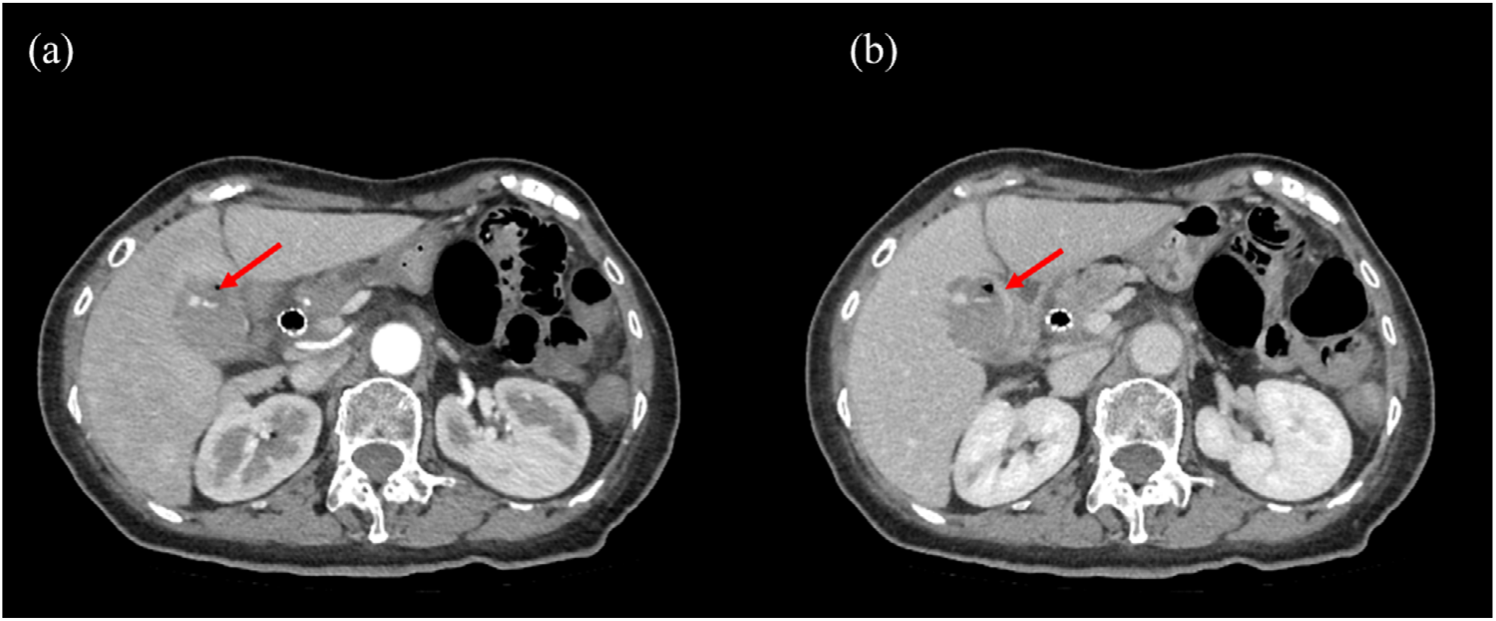
(a) Contrast‐enhanced computed tomography (arterial phase) showing rupture of a cystic artery pseudoaneurysm (arrow). (b) Contrast‐enhanced computed tomography (equilibrium phase) showing rupture of a cystic artery pseudoaneurysm (arrow).

Seven days after SEMS placement, she presented to the emergency department after two episodes of copious hematochezia accompanied by mild upper abdominal pain and fever. Vital signs at presentation included a temperature of 37.0°C, a heart rate of 87 beats per minute, blood pressure of 137/84 mmHg, a respiratory rate of 24 breaths per minute, and SpO_2_ of 95% on room air. Physical examination revealed jaundice, but no abdominal tenderness or Murphy's sign. Laboratory tests showed anemia, elevated inflammatory markers, and elevated liver enzymes (Table 1). Contrast‐enhanced computed tomography revealed a cystic artery pseudoaneurysm with extravasation of contrast into a blood‐filled gallbladder (Figure 2). The cholecystitis observed during the previous hospitalization had improved. The fully covered SEMS remained in the initial location, and no biliary or cystic duct obstruction was observed.

**TABLE 1:** Laboratory data at admission for cystic artery pseudoaneurysm rupture.

White blood cell count	16.83	10^3^/μL
Red blood cell count	3.04	10^6^/μL
Hemoglobin	9.3	g/dL
Platelet count	386	10^3^/μL
Total bilirubin	1.4	mg/dL
γ‐Glutamyl transpeptidase	385	U/L
Alkaline phosphatase	1519	U/L
Aspartate aminotransferase	234	U/L
Alanine aminotransferase	233	U/L
Blood urea nitrogen	11	mg/dL
Creatinine	0.52	mg/dL
C‐reactive protein	3.07	mg/dL

**FIGURE 3:**
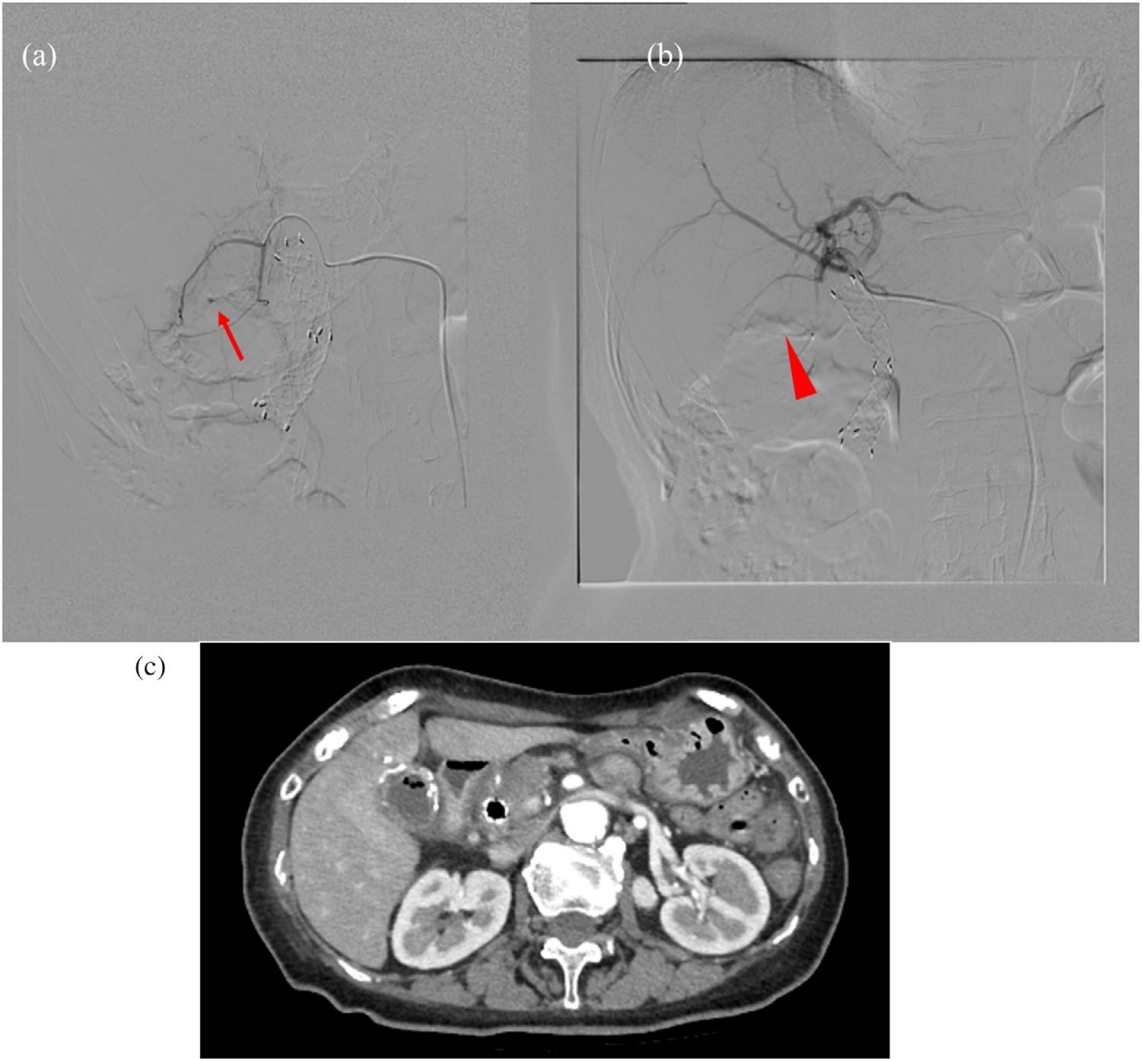
(a) Cystic artery pseudoaneurysm on angiography (arrow). (b) Blood flow to cystic artery pseudoaneurysm disappeared after transcatheter arterial embolization (arrowhead). (c) Contrast‐enhanced computed tomography after transcatheter arterial embolization confirmed the absence of blood flow to the deep cystic artery.

Emergent angiography was performed. A micro‐catheter (Estream 1.7VA; Toray Medical Co., Ltd.) was advanced from the right femoral artery to the cystic artery. In cystic artery angiography, contrast agent leakage was observed, so TAE was performed with 1:2 mixtures of *N*‐Butyl‐2‐cyanoacrylate and ethiodized oil. Angiography after embolization showed that all three visualized branches of the deep cystic artery were occluded, but the superficial cystic artery remained intact. No active bleeding was observed after TAE. Contrast‐enhanced computed tomography 1 week after TAE showed that the cystic artery pseudoaneurysm had disappeared, and no ischemia of the gallbladder wall was observed (Figure 3). The patient's jaundice and elevated liver enzymes resolved after TAE, without requiring biliary drainage. She was discharged 11 days later without the need for any blood transfusions. She had no signs of cholecystitis or re‐bleeding on follow‐up 1 month after TAE.

## DISCUSSION

To our knowledge, there are no reports of cystic artery pseudoaneurysm rupture after biliary SEMS placement for obstructive jaundice due to pancreatic cancer in the existing literature. Obstruction of the lower common bile duct can not only lead to cholangitis but can also give rise to concurrent cholecystitis. Pseudoaneurysms formed after SEMS placement in any location is reported in 1.2% of cases.^3^ The timing of pseudoaneurysm formation after SEMS placement ranges from 5 days to 2 years.^3^, ^5^ As patients with pseudoaneurysm after SEMS placement have a history of chemotherapy or chemoradiotherapy, such antineoplastic treatments may play a role in pseudoaneurysm formation.^3^ Endoscopic ultrasound‐guided hepaticogastrostomy is increasingly performed as an alternative to ERCP for biliary drainage, and pseudoaneurysm rupture after endoscopic unltrasound‐guided hepaticogastrostomy has also been reported.^6^

The typical symptoms of hemobilia are known as Quincke's triad, which consists of jaundice, upper quadrant abdominal pain, and upper gastrointestinal bleeding. However, all three symptoms are observed in only 22% of the cases.^7^ In this case, although jaundice and upper abdominal pain were mild, all three symptoms were present. The causes of hemobilia are mainly iatrogenic, including percutaneous interventions, endoscopic interventions, and surgery.^7^ Non‐iatrogenic causes of hemobilia include malignant disease, portal biliopathy, chronic ductal obstruction, and infection. The most common cause of hemobilia after ERCP is post‐endoscopic sphincterotomy bleeding, with a reported frequency of 2.8%.^8^ Although pseudoaneurysms are rarer than post‐endoscopic spincterotomy bleeding, pseudoaneurysms are a serious complication and often require emergent procedures.

Pseudoaneurysms related to SEMS are formed due to mechanical damage caused by the stent edge or due to necrosis of the arterial wall caused by the pressure of SEMS. Therefore, the mechanical properties of SEMS may affect pseudoaneurysm formation. While pseudoaneurysms after SEMS placement are most commonly found in the right hepatic artery near the distal end of the SEMS, the gastroduodenal and posterior superior pancreaticoduodenal arteries can also be affected.^3^, ^9^ In this case, a pseudoaneurysm formed in the cystic artery, which was not in direct contact with SEMS. However, the stent may have exerted pressure on extrabiliary structures including the inflamed gallbladder, playing a role in pseudoaneurysm formation and subsequent rupture. The possibility of repeated mechanical irritation to the artery due to respiratory movement of the stent also cannot be ruled out. There have also been reports of an association between anticoagulants and pseudoaneurysm formation, and rivaroxaban may have played a role in pseudoaneurysm formation in this case.

Reports on cystic artery pseudoaneurysms are scarce. Fujimoto et al. reviewed 51 cases of cystic artery pseudoaneurysm.^4^ The development of cystic artery pseudoaneurysm was associated with gallbladder inflammation in all cases. Cystic artery pseudoaneurysm was associated with gallstones in 91% of cases. The pseudoaneurysm ruptured in 86% of cases, with intraperitoneal bleeding observed in 18% of cases. Treatment for cystic artery pseudoaneurysm included cholecystectomy in 75% of cases and TAE in 53% of cases. Saito et al. reported a case of ruptured cystic artery pseudoaneurysm after ERCP.^10^ In that case, a cystic artery pseudoaneurysm ruptured 3 months after plastic stent placement during endoscopic transpapillary gallbladder drainage. The authors speculated that long‐term placement of the plastic stent may have stimulated the nearby cystic artery, leading to pseudoaneurysm formation.

In our case, the patient did not have gallstones or direct stimulation to the gallbladder from SEMS placed in the common bile duct. However, an enlarged gallbladder with wall thickening was observed due to malignant distal biliary obstruction. After SEMS placement, the gallbladder shrank in size, but we hypothesize that cholecystitis led to the weakening of the gallbladder wall and that the cystic artery wall was exposed to inflammation, leading to arterial wall damage and pseudoaneurysm formation. Although cystic artery pseudoaneurysms can be treated curatively by cholecystectomy, we performed TAE without cholecystectomy due to the advanced stage of the patient's pancreatic cancer and the performance status of the patient. Caution is required after TAE of the cystic artery, as decreased blood flow can lead to gangrenous cholecystitis. In our case, we were able to avoid this feared complication by ensuring the superficial cystic artery was preserved.

In conclusion, rupture of cystic artery pseudoaneurysm should be raised as a differential diagnosis for hemobilia after SEMS placement, particularly in cases accompanied by inflamed gallbladders.

## CONFLICT OF INTEREST STATEMENT

The authors declare no conflict of interest.
